# Supplementary material for: Adaptation to a host-associated lifestyle is associated with convergent loss of flagella-related genes in Pseudomonadota
Source: BMC Genomics. 2026 Apr 9;27:366. doi: 10.1186/s12864-026-12835-3 (PMC13063649; doi:10.1186/s12864-026-12835-3)
Supplement: Supplementary file 1 — Supplementary Figure 1. [file 12864_2026_12835_MOESM1_ESM.pdf]

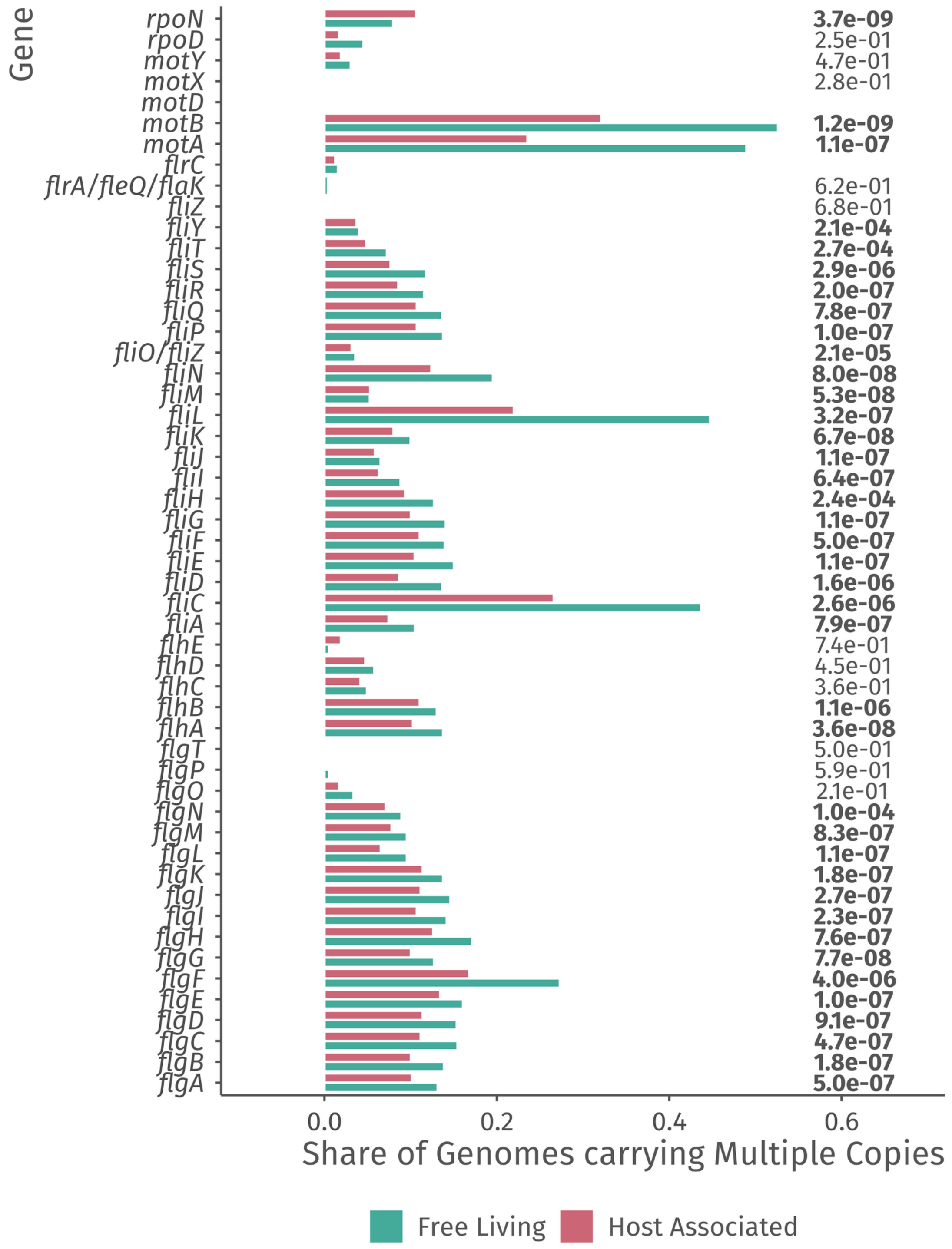

Supplementary Figure 1. Host associated bacteria harbor less copies of supernumerary genes

Share of genomes, divided into the free-living and host-associated groups, carrying more than one copy of each gene. False discovery rates from the phylogenetic Poisson regression on the right side of graph show the significance of the effects of host-association on the number of copies of each gene. Multiple copies are consistently detected for the filament gene *fliC*, the motor genes *motA* and *motB,* and the switch gene *fliL,* with significant differences between free-living and host-associated bacteria.
